# An Unusual Case of Cystic Fibrosis Associated* Pneumocystis jiroveci* Pneumonia in an Infant

**DOI:** 10.1155/2016/9206707

**Published:** 2016-12-13

**Authors:** Ravinder Kaur, Priyanka Katariya, Megh Singh Dhakad, Bhanu Mehra, Urmila Jhamb, A. P. Dubey

**Affiliations:** ^1^Department of Microbiology, Lady Hardinge Medical College and Associated Hospitals, New Delhi 110001, India; ^2^Department of Microbiology, Maulana Azad Medical College and Associated Lok Nayak Hospitals, New Delhi 110002, India; ^3^Department of Pediatrics, Maulana Azad Medical College and Associated Hospitals, New Delhi 110002, India

## Abstract

*Pneumocystis jiroveci* pneumonia (PJP) is one of the major infections in patients with impaired immunity. The entity is common in HIV-seropositive individuals but quite very rare in HIV-seronegative individuals especially children. We report here a case of 16-week-old HIV-seronegative infant with chief complaint of chronic cough of one month of evolution. Sweat chloride test for diagnosis of cystic fibrosis was positive. Bronchoalveolar lavage (BAL) fluid was collected and* Pseudomonas aeruginosa* was isolated on culture. Empirical antibiotic regimen comprising ceftriaxone and azithromycin was initiated that was switched to meropenem as per antimicrobial susceptibility report, but the patient did not improve. Subsequently, an immunofluorescence staining of BAL fluid was performed and* P. jiroveci* cysts were detected. Following a laboratory confirmation of* Pneumocystis* pneumonia, cotrimoxazole was added and the clinical condition of the patient significantly improved. This is an unusual case wherein unsuspected PJP occurred and since signs and symptoms of the patient persisted even after the initiation of antimicrobial therapy for* Pseudomonas* infection and resolved only after treatment for PJP was started, it suggests a causative role of* P. jiroveci* rather than colonization/contamination.

## 1. Introduction

*Pneumocystis jiroveci*, formerly called* Pneumocystis carinii*, is an ascomycetous fungus that causes an opportunistic pneumonia.* Pneumocystis jiroveci* pneumonia (PJP) is one of the major infectious complications in immunocompromised patients, whether those living with human immunosuppressive virus (HIV) or with non-HIV immunosuppressive states [1]. PJP was first described by Otto Jirovec in European infants with pneumonia [2]. The mortality rates of PJP in patients with or without AIDS remain high at 30%–60% [3]. The clinical diagnosis of PJP is complicated by the nonspecific signs and symptoms and also due to lack of reliable culture system for the organism [2].* Pneumocystis* infections are almost always limited to the lungs and microorganism cannot be cultured reliably outside the lungs [2]. Here we are presenting a case of unsuspected PJP in an 16-week-old HIV-seronegative female child with complaint of chronic cough of one month of evolution.

## 2. Case History

A 16-week-old female child was admitted to a tertiary care hospital in New Delhi with complaint of chronic cough of one month of evolution. Birth history revealed an uncomplicated pregnancy with a spontaneous, full term normal vaginal delivery at home through clear liquor. Prelacteal feeds were started on day 1 and breast feeding started on day 2 of life. The baby was doing well till day 6 when the mother noticed that the infant developed cough. The cough was more on lying down position and was aggravated after feed. The patient was given some oral medication but cough persisted and continued to increase. Cough became pronounced at one month of evolution and progressively worsened. Her stools were noted to be frequent and runny. There was history of respiratory distress with chest retractions along with suck-rest-suck cycle and cyanosis. No family history for immunodeficiency disorders, cystic fibrosis, or respiratory diseases could be elicited. In addition, history of contact with an individual at high risk for PJP disease was negative.

Physical examination on admission revealed a weight of 3.7 kg (57.81%), length of 52 cm (92.05%), and head circumference of 37 cm (78.2%). The respiratory rate was 60/minute and heart rate was 140/min. Haemoglobin oxygen saturation was 98%. The infant was thin and pale. The skin was normal. There was no oral thrush. Chest examination revealed subcostal retractions and mild hyperinflation with occasional crepitations. Examination of cardiovascular system revealed early systolic murmur. The abdomen was soft and nontender without hepatosplenomegaly or palpable masses.

Haemoglobin was 12.9 g/L, TLC was 14900/cumm (66% neutrophils, 29% lymphocytes, and 5% monocytes), and platelet count was 3.96 lakhs/cumm. Sweat chloride and sodium were 102 mmol/L and 90 mmol/L, respectively (positive for cystic fibrosis). Computed Tomography (CT) of chest revealed multiple ill-defined nodular opacities, seen in bilateral lower lobes with areas of ground glass opacities in the left lobe. Cardiac Echo revealed ACHD/OS ASD/RA/RVD with left-right shunt. An ELISA for anti-HIV antibodies was negative.

## 3. Materials and Methods

BAL fluid was collected with a 3.5 mm diameter, flexible fibreoptic bronchoscope advanced into the segmental bronchi in the lower lobes. 10 cc aliquots of sterile normal saline were instilled into separate segments of the right lower lobe. After collection, the BAL sample was sent to the Mycology laboratory, department of Microbiology, for direct microscopic examination and fungal culture. Cytospin centrifuge smears of BAL were prepared and the presence of* P. jiroveci* was determined by staining the slide with a specific fluorescein isothiocyanate-labelled monoclonal antibody that recognizes* P. jiroveci* cyst and trophozoites. Immunoflourescent staining was done by using Merifluor Pneumocystis kit (Meridian Bioscience, Inc., Cincinnati, Ohio) based on the principle of direct fluorescent microscopy for detection of Pneumocystis antigen (both cyst and trophozoite forms).

## 4. Results

**Figure 1:**
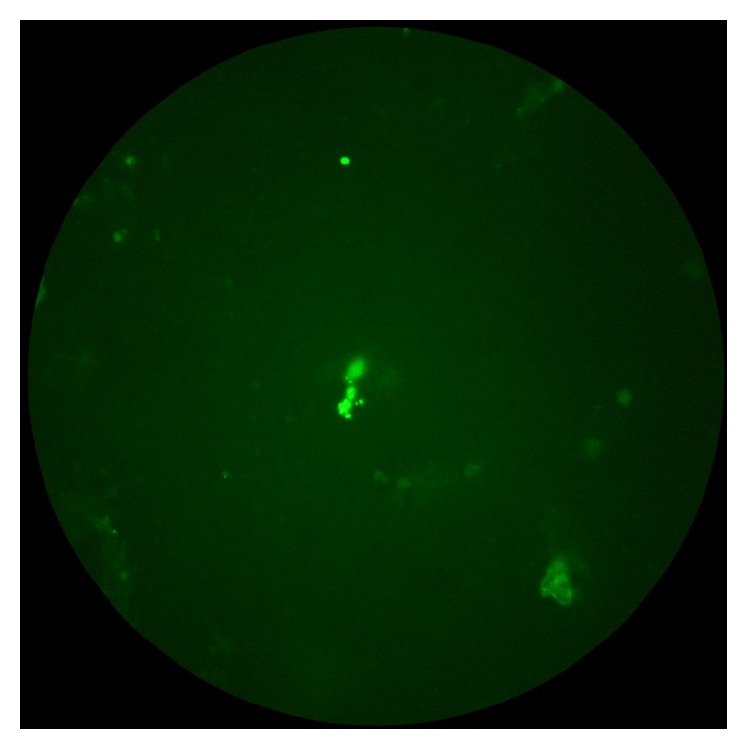
*P. jiroveci* in BAL fluid.

Bronchoscopy revealed no anatomical abnormality in the airways. The lavage fluid was thick and copious. Cytology revealed presence of neutrophils, eosinophils, and alveolar cells. A direct microscopic examination of BAL fluid by Gram stain revealed only pus cells; no acid fast bacilli were seen by Ziehl Neelsen stain. On routine aerobic culture,* Pseudomonas aeruginosa* was isolated which was sensitive to amikacin, gentamycin, tobramycin, meropenem, and colistin. When the patient did not improve despite 2 weeks of meropenem therapy and a subsequent BAL fluid immunofluorescence staining revealed* P. jiroveci* cysts (Figure 1), cotrimoxazole was started and continued over 2 weeks and resulted in complete clinical recovery of the patient.

## 5. Discussion

*Pneumocystis jiroveci* was initially classified as a protozoan based on the histologic characteristics of its trophozoite and cyst forms and its treatment response with the antiprotozoan medication pentamidine [4]. In 1988,* P. jiroveci* was phylogenetically reclassified as it was found to have a homology to ascomycetous fungi [4]. It causes an opportunistic pneumonia in immunocompromised hosts who usually have T-cell defects and even in those with B-cell deficiencies. The pneumonia is characterized by proliferation of cysts, sporozoites, and trophozoites within the alveoli and is associated with proteinaceous exudate into the airspaces and surrounding interstitial inflammation.* P. jiroveci* usually does not cause clinically apparent pneumonia in individuals with intact immunity. The mechanism for transmissibility and the normal distribution of* P. jiroveci* among humans and other species are incompletely understood [5]. The sites of infection and route of entry in humans are not known, though presumably the lung is the major site. It is not known whether infected individuals transmit the disease or whether there is a life cycle outside of humans responsible for acquisition of new* P. jiroveci* infections [6]. Epidemiologic evidence as well as evidence in animal models suggest that* P. jiroveci* can persist in a latent state from early childhood, with subsequent reactivation when host immunity wanes. The location of colonization and the mechanism for reactivation have not been established, in part due to the previous absence of sensitive methods of detection [7]. Evidence from animal models suggests that the airborne route is an important mode of transmission [7].

In a 10-year study from Poland on serum samples collected from 5223 children up to 10 years of age with respiratory tract infections, specific anti-*Pneumocystis* antibodies were detected in 68.7% of the examined children (68.49% with IgG and 2.85% with IgM antibodies). The highest percentages of IgM and IgG positive samples were encountered in children in the second half of the first year of life (73%) [8].* P. jiroveci* colonization may occur among cystic fibrosis (CF) patients because of their underlying pulmonary disease [9]. A few published studies carried out in Europe have evaluated the prevalence of* Pneumocystis* colonization in patients with CF and found it to range from 1.3 to 21.6% [10]. A study from Brazil on 34 CF patients (range: 1 to 35 years; median age: 11 years) found this colonization rate to be 38.2% [11]. A French study on 104 CF patients (median age: 24 years) showed a percentage of about 12.5% in this regard [12]. The differences in colonization rates might be related to the different types of clinical samples that were analyzed. It has been suggested that BAL samples provide the highest sensitivity for the diagnosis of PCP by molecular methods [11].

The evolution of* P. jiroveci* colonization in CF patients is largely unknown [10]. It is normally believed that* Pneumocystis* colonization in CF patients does not progress to overt pneumonia as CF patients normally have an intact immune system. Marked immunosuppression is the crucial factor for development of pneumonia [10]. There are no definite reports of* P. jiroveci* causing infection in CF patients. In immunocompetent individuals who have chronic lung disease,* Pneumocystis* colonization could provoke mild or moderate respiratory illness, with the pathogen acting as a comorbidity factor [10, 13]. Low levels of* Pneumocystis* in the lungs may stimulate pulmonary inflammation and may play a role in the development of lung diseases [14].

In our case, the signs and symptoms of the patient persisted even after the initiation of specific antimicrobial therapy for* Pseudomonas* infection and resolved only after treatment for* P. jiroveci* was instituted indirectly suggesting infection with* P. jiroveci* rather than colonization.* P. jiroveci* infection is known to present as interstitial pneumonitis in infants who are severely malnourished [6]. Presence of* P. jiroveci* cysts in BAL fluid along with associated clinical and radiological findings signifies infection over colonization of* P. jiroveci* in the patient. There is paucity of data to date regarding colonization rates with* P. jiroveci* in CF, using BAL and histologic and/other techniques to identify the potential pathogen. However, in infants with CF associated lung disease not improving on antimicrobial therapy based on susceptibility studies, BAL fluid is an important respiratory sample and should be examined for possible* P. jiroveci* infection. One needs to further dwell on the fact that* P. jiroveci* infection may worsen the CF associated lung disease by invasion/immune stimulation and whether BAL should be routinely analyzed in all cases of CF or in more complicated presentations of the same CF.
